# Hemodynamic Pattern Recognition During Deception Process Using Functional Near-infrared Spectroscopy

**DOI:** 10.1007/s40846-016-0103-6

**Published:** 2016-03-10

**Authors:** Roberto Vega, Ana G. Hernandez-Reynoso, Emily Kellison Linn, Rita Q. Fuentes-Aguilar, Gildardo Sanchez-Ante, Arturo Santos-Garcia, Alejandro Garcia-Gonzalez

**Affiliations:** Tecnológico de Monterrey, Campus Guadalajara, 45201 Monterrey, Mexico; Department of Electrical Engineering and Computer Science, Massachusetts Institute of Technology, Cambridge, 02139 USA

**Keywords:** Functional near-infrared spectroscopy (fNIRS), Deception detection, Hemodynamic activity, Pattern recognition

## Abstract

Deception is considered a psychological process by which one individual deliberately attempts to convince another person to accept as true what the liar knows to be false. This paper presents the use of functional near-infrared spectroscopy for deception detection. This technique measures hemodynamic variations in the cortical regions induced by neural activations. The experimental setup involved a mock theft paradigm with ten subjects, where the subjects responded to a set of questions, with each of their answers belonging to one of three categories: Induced Lies, Induced Truths, and Non-Induced responses. The relative changes of the hemodynamic activity in the subject’s prefrontal cortex were recorded during the experiment. From this data, the changes in blood volume were derived and represented as false color topograms. Finally, a human evaluator used these topograms as a guide to classify each answer into one of the three categories. His performance was compared with that of a support vector machine (SVM) classifier in terms of accuracy, specificity, and sensitivity. The human evaluator achieved an accuracy of 84.33 % in a tri-class problem and 92 % in a bi-class problem (induced vs. non-induced responses). In comparison, the SVM classifier correctly classified 95.63 % of the answers in a tri-class problem using cross-validation for the selection of the best features. These results suggest a tradeoff between accuracy and computational burden. In other words, it is possible for an interviewer to classify each response by only looking at the topogram of the hemodynamic activity, but at the cost of reduced prediction accuracy.

## Introduction

Deception is considered a psychological process by which one individual deliberately attempts to convince another person to accept as true what the liar knows to be false [1]. Deception detection mechanisms using scientific techniques and technologies are important because of their applications to business, security, and legal problems; however, this is a challenging task, and the current technical and methodological methods for detecting intentional deceptions are inadequate [2].

In general terms, there are two approaches to deception detection, namely those based on psychophysiological and neurological mechanisms, respectively. Psychophysiology mechanisms are subtle physiological changes related to lying, such as changes in respiration, skin surface temperature, or heart rate. Among the technologies based on these mechanisms are the polygraph, electrogastrogram, vital signs measurements, facial expression recognition, thermal imaging, and voice stress analysis.

Methods based on neurological mechanisms include electroencephalography, magnetoencephalography, positron emission tomography, functional magnetic resonance imaging, and functional near-infrared spectroscopy (fNIRS). These technologies allow the observation of the neurophysiological activity of the brain, and thus can be used to identify the brain processes related to deception. A complete description of each technique, and its potential as a lie detector, is given elsewhere [2]. The present study proposes the use of fNIRS for detecting deception. fNIRS is selected due to its low cost, portability, fair spatial resolution, and non-ionizing brain imaging ability [3].

### Functional Near-infrared Spectroscopy

The biological basis behind fNIRS is a process known as neurovascular coupling. The coupling between neuronal activity and the local control of blood flow and oxygenation (called hemodynamics) in the brain allows the measurement and localization of neuronal activity. An increase in brain activity causes an increase in oxygen consumption due to an increased metabolic demand. This, in turn, causes changes in the concentrations of oxyhemoglobin and deoxyhemoglobin in the blood vessels, which translates into an increase of local blood flow after a delay of approximately 2 s [4].

fNIRS is a field-deployable non-invasive functional optical brain monitoring technology that measures hemodynamic variations in the cortical regions induced by sensory, motor, or cognitive activation [3, 5]. It relies on the fact that near-infrared light can penetrate through the human scalp and skull, reaching the cortex [6]. However, the raw signals obtained from an fNIRS device contain not only information about the hemodynamic response of the brain, but also information about physiological signals such as heart rate and respiration [7].

Light in the near-infrared spectrum in the range of 730–950 nm can propagate several centimeters inside tissues [6]. Light in this spectrum is diffused through the intact scalp and skull and can be used for tracing hemoglobin concentration changes within the brain [3]. Oxygenated and deoxygenated hemoglobin (*HbO*_2_ and *Hb*, respectively) exhibit characteristic optical properties in this wavelength range. The specific wavelength selection is an optimization problem of maximizing the discrimination between oxyhemoglobin and deoxyhemoglobin concentrations while satisfying the following two conditions [6]:

1. One wavelength must be greater than 780 nm, and the other must be lower than 780 nm.
2. Crosstalk between oxyhemoglobin and deoxyhemoglobin must be as low as possible.

The measured changes in concentration of *Hb* and *HbO*_2_ are relative to an initial measurement (baseline) [7]. It is important to state that when recording the baseline, non-evoked signals associated with neuromuscular coupling are also recorded; these signals contribute to the variability of the fNIRS signal [6]. Averaging each signal in the time window in which the baseline is recorded diminishes this effect. A full description of the basis of the fNIRS technique as well the equipment characteristics (temporal and spatial resolution, data acquisition, etc.) can be found elsewhere [3].

### fNIRS as Deception Detection Technique

There is increasing interest in fNIRS as a deception detection method [8–12]. Tian et al. [9] reported that there are significant changes in hemoglobin concentration associated with deceptive responses relative to a baseline, compared with differences not statistically significant when subjects are telling the truth. They averaged the relative changes in oxyhemoglobin and deoxyhemoglobin among eleven subjects and showed the characteristic deceptive and truthful behaviors. Their proposal is to create a topographic representation based on the average behavior during the interrogation, but the user does not apply the current mapping during each question to detect deception.

Kozel et al. [12] reported that the prefrontal cortex has a greater activation during deception, especially in the left dorsolateral and right anterior prefrontal cortices. These results showed that fNIRS obtains similar results to those found by functional magnetic resonance imaging studies of deception. Ding et al. [10] explored the involvement of the prefrontal brain regions in spontaneous deception. They found that when a subject is lying (either in spontaneous or instructed deception), the left superior frontal gyrus presents more activity. Finally, Hu et al. [11] used support vector machines (SVMs) to classify the responses of eight subjects in deception and truth-telling scenarios.

Studies using fNIRS to detect lies have focused on the mathematical and computational analysis of the signals, making it difficult for the users to read numerical results. The present work proposes a methodology that allows the classification of a given answer as a lie or a truth, and compares two approaches to achieve this task: a visual one, in which an interviewer classifies each answer using only a false color topogram that maps the hemodynamic changes in the prefrontal cortex of an interviewee, and an automatic one based on an SVM classifier.

## Materials and Methods

### Equipment

Hemodynamic changes were acquired using an fNIRS Model 1100 Imager by fNIR Devices LLC, a non-invasive oxygenation and blood volume trend imager, and its software Cognitive Optical Brain Imaging Studio (COBI Studio). The system has a flexible fNIRS sensor pad with four light sources at two wavelengths (730 and 850 nm), designed to monitor dorsal and inferior cortical areas underlying the forehead [3]. There are ten sensors that measure the photons reflected back from the tissues at a sampling rate of 2.004 Hz for each channel, with a temporal resolution of 500 ms per scan and approximately 1.25 cm of penetration depth. This sensor allows the monitoring of the dorsal and inferior frontal areas underlying the forehead [3]. Thus, there are 16 voxels, each one deriving in two discrete time signals, which are proportional voltages to the absorption at each wavelength.

### Subjects and Experimental Setup

The objective of the experiment was to discriminate between deceptive and truthful behavior by measuring brain activity changes in the prefrontal cortex using fNIRS. The brain activity of ten subjects was measured while they answered an interview composed of 30 questions. The procedures followed were in accordance with the Helsinki Declaration of 1975, as revised in 2004. The responses of the participants were classified into four categories represented by different values of variable *m* as follows:

1. Induced lies (*m* = 1)
2. Induced truths (*m* = 2)
3. Non-induced lies (*m* = 3)
4. Non-induced truths (*m* = 4)

For Induced Lies and Induced Truths, the subject was explicitly asked to respond with lies or truths, and for Non-Induced Lies and Non-Induced Truths, the subject answered the questions without instruction to either lie or tell the truth [8]. The experiment involved a Guilty Knowledge Test (GKT), based on a previously reported one [9]. The GKT is summarized as follows:

1. The participant enters the room where the experiment takes place and takes a seat in front of a closed box.
2. The participant is instructed to take one of two objects, A or B, from the box when the investigator leaves the room, and hide it.
3. The fNIRS equipment is placed on the participant’s forehead and calibrated according to the manufacturer’s specifications.
4. The participant is asked to relax for 10 min, and then the baseline is measured.
5. The signal recording is started.
6. The interview, which is divided into three stages, begins. In the first stage, participants are asked to respond as if they had not taken any object (Non-Induced responses). In the second stage, participants are asked to respond only with lies (Induced Lies), while in the third stage, they are asked to respond only with truths (Induced Truths). In each stage, the participant answers 10 questions.
7. The signal recording ends.
8. The participant is asked to answer all the previous questions truthfully at the end of the interview. These answers are taken as the ground truth.

Unlike in the work by Tian [9], the subjects were not trained and the answers were verbal instead of given via a keyboard.

### fNIRS Signal Preprocessing

**Fig. 1:**
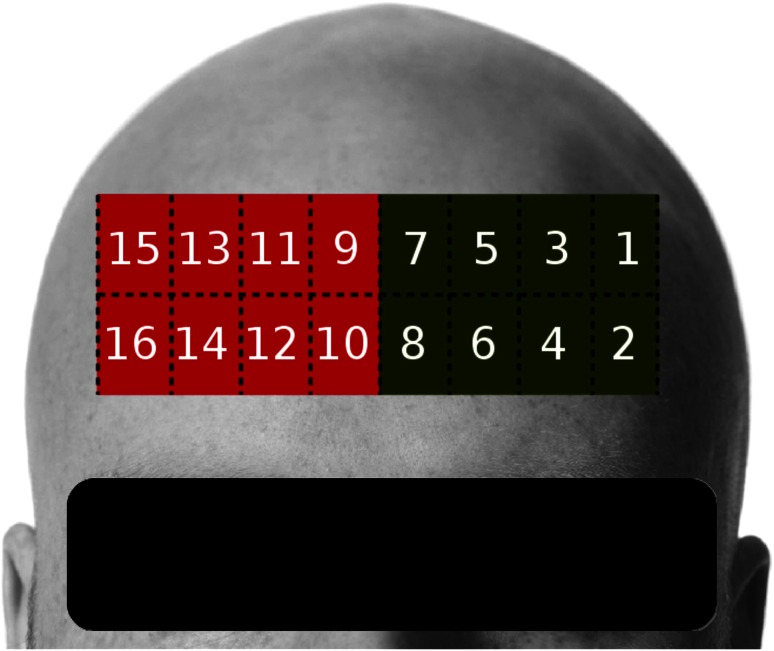
Voxel distribution of device containing 16 voxels. The voxels can be grouped into those corresponding to brain’s left hemisphere (*green*) and right hemisphere (*red*)

The fNIRS device outputs light intensity data as a voltage signal for two wavelengths. The total number of raw signals is 2*i*, where *i* is the number of voxels in the device. In the fNIRS context, a voxel is a two-dimensional structure that covers a certain area of the forehead. Each voxel outputs two voltage signals, one for each wavelength. Figure 1 shows the voxel distribution of a device containing 16 voxels.

These signals are filtered using a low-pass finite impulse response (FIR) filter with a cutoff frequency of 0.14 Hz. [13]. A detailed procedure for the design of a FIR filter using the Hamming window can be found elsewhere [14].

After the signal had been filtered, the relative changes in concentration of oxyhemoglobin and deoxyhemoglobin versus time were estimated by measuring the change in optical density (*OD*) using the modified Beer-Lambert equation [7]:

1 $$\Delta OD\left( k \right) = \log \frac{{I_{b} }}{I\left( k \right)} = \alpha_{Hb} \Delta C_{Hb} \left( k \right) + \alpha_{{HbO_{2} }} \Delta C_{{HbO_{2} }} \left( k \right)$$

where Δ*OD*(*k*) is the change in optical density (μmol/L) at the current sample *k*, *I*_*b*_ is the light intensity measurement at the baseline, *I*(*k*) is the light intensity measure at a given sample *k*, α_*Hb*_ and $\alpha_{{HbO_{2} }}$ are the molar extinction coefficients, and Δ*C*_*Hb*_(*k*) and $\Delta C_{{HbO_{2} }}$(*k*) are the relative changes (μmol/L) in *Hb* and *HbO*_*2*_, respectively. It is also possible to estimate the relative changes in blood volume (ΔB(*k*)) and blood oxygenation (ΔO(*k*)) as follows [7]:

2 $$\begin{aligned} \Delta B\left( k \right) = \Delta C_{{HbO_{2} }} \left( k \right) + \Delta C_{Hb} \left( k \right) \hfill \\ \Delta O\left( k \right) = \Delta C_{{HbO_{2} }} \left( k \right) - \Delta C_{Hb} \left( k \right) \hfill \\ \end{aligned}$$

### Question-Level Processing

**Fig. 2:**
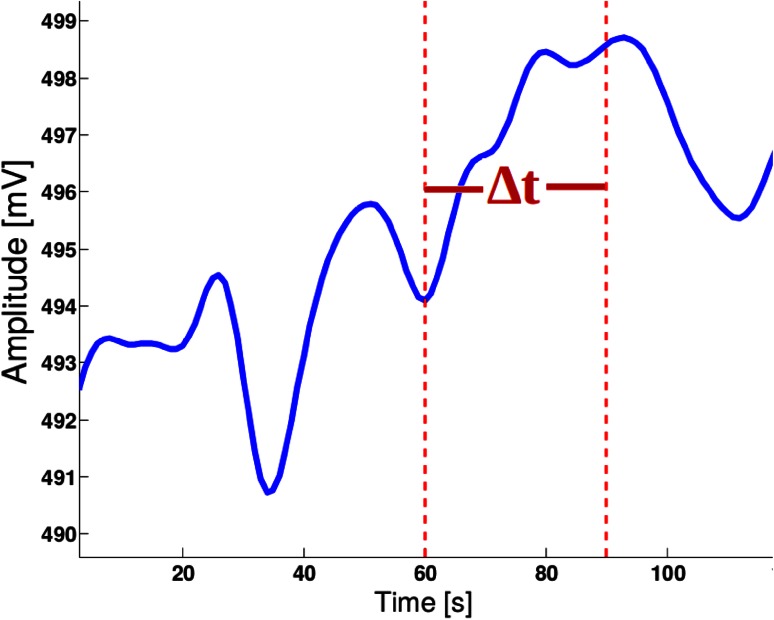
The time window of length Δ_t_ following a stimulus is analyzed for each voxel

According to a previous study [15], the average hemodynamic peak time, after telling either a lie or a truth, is within 1.6 s. Therefore, the relative changes in oxygenation and blood volume were computed (using Eq. (2)) for each voxel in a time window of 5 s immediately following a stimulus (the end of a question), as shown in Fig. 2. Then, the average of the relative changes in blood volume at each voxel ($\overline{\Delta B}_{v}^{q}$) along this time period was computed as:

3 $$\overline{\Delta B}_{v}^{q} = \frac{1}{n}\sum\limits_{k = 1}^{n} {\Delta B_{v}^{q} \left( k \right)}$$

where *n* is the number of samples in the 5-s window and Δ*B*_*v*_^*q*^(*k*) is the change in blood volume for question *q* = 1, 2, …, *Q*, in voxel *v* = 1, 2, …, *V,* at sample *k. Q* is the total number of questions and *V* is the total number of voxels. Analogously, the average of the relative changes in oxygenation at each voxel ($\overline{\Delta O}_{v}^{q}$) was computed.

### Class-Level Processing

After $\overline{\Delta B}_{v}^{q}$ and $\overline{\Delta O}_{v}^{q}$ had been computed, all the questions for a particular subject were grouped into the classes described in Sect. 2.1. Then, the mean of each class by voxel $\overline{\Delta B}_{v}^{m}$ was computed as:

4 $$\overline{\Delta B}_{v}^{m} = \frac{1}{{Q_{m} }}\sum\limits_{q = 1}^{{Q_{m} }} {\overline{\Delta B}_{v}^{q} }$$

where *Q*_*m*_ is the number of questions that belong to class *m* (*Q*_*m*_ ⊂ *Q*). Analogously, the mean value of class *m* at voxel *v* for the relative changes in oxygenation $\overline{\Delta O}_{v}^{m}$ was computed.

### Feature Selection

The objective of this stage is to reduce the number of information that a human observer has to analyze to perform visual classification. After $\overline{\Delta B}_{v}^{q}$, $\overline{\Delta O}_{v}^{q}$, $\overline{\Delta B}_{v}^{m}$, and $\overline{\Delta O}_{v}^{m}$ are computed for every subject independently, the most relevant voxels *v*_*r*_ are selected to discriminate among classes according to the following criteria:

- Heuristic criterion: $\left| {\overline{\Delta B}_{v}^{m = 1} - \overline{\Delta B}_{v}^{m = 2} } \right| \ge 1$, for *v* = 1,2,…,V. If no voxel meets this condition, take the two voxels with the greatest difference. If only one voxel meets this condition, take it along with the feature that shows the second greatest difference. Analogously, use the same criteria for $\left| {\overline{\Delta B}_{v}^{m = 3} - \overline{\Delta B}_{v}^{m = 4} } \right| \ge 1$.

- Non-parametric criterion: ∀*m* ∊ *M* = {1, 2, 3, 4} define $S_{v}^{m} = \left\{ {\overline{\Delta B}_{v}^{q = j} ,\overline{\Delta B}_{v}^{q = j + 1} , \ldots ,\overline{\Delta B}_{v}^{q = L} } \right\}$ for *q* = {*j,j* + 1,…,*L*} that belong to class *m*, and *v* = 1, 2, …,*V.* Compare the boxplots of *S*_*v*_^*m*=1^ versus *S*_*v*_^*m*=2^ and *S*_*v*_^*m*=3^ versus *S*_*v*_^*m*=4^. Select the voxels with no overlap in the interquartile range of their boxplots.
- Parametric criterion: Select the voxels with statistically significant differences (α = 0.1, two-tailed *t* distribution) in $\left( {\overline{\Delta B}_{v}^{m = 1} - \overline{\Delta B}_{v}^{m = 2} } \right)$,$\left( {\overline{\Delta B}_{v}^{m = 3} - \overline{\Delta B}_{v}^{m = 4} } \right)$ and $\left( {\overline{\Delta B}_{v}^{m = 1,2} - \overline{\Delta B}_{v}^{m = 3,4} } \right)$.

**Fig. 3:**
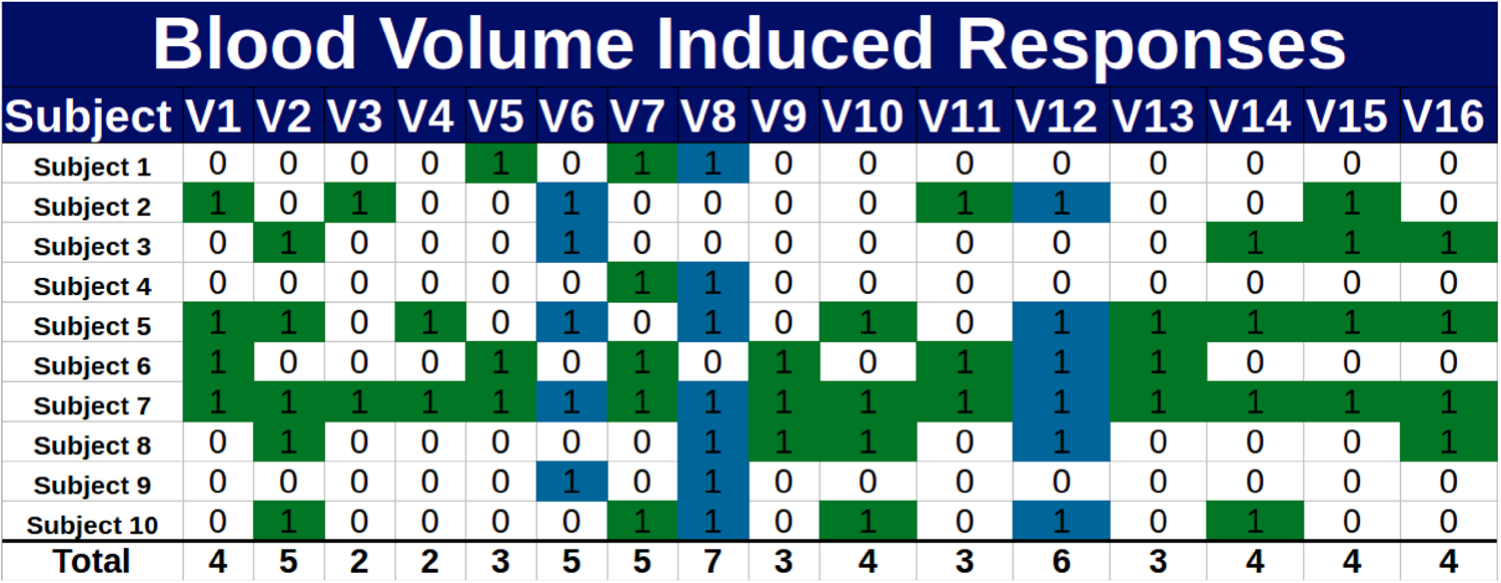
Selection of most frequent voxels used to discriminate between induced truths and induced lies using the difference between means. In *green* are all voxels that met the selection criteria. In *blue* are the voxels that met the criteria and were selected

The voxels that met at least one of these three conditions were selected as candidate features. Matrices of all the subjects, marked with a “1” for candidate features and a “0” for non-candidate features, were then created. A total of 14 matrices were created for both relative change in blood volume and relative change in oxygenation (7 for each one). The group of voxels that appeared most frequently was selected, as shown in Fig. 3. The objective was to find the smallest set of voxels containing at least one *v*_*r*_ for each subject. The features selected were $\overline{\Delta B}_{v}^{q}$ and $\overline{\Delta O}_{v}^{q}$ for the most frequent voxels.

### Classification

The selected features were used to classify each question into one of the possible classes. The performance of visual classification was compared with that of automatic classifiers using the features found by each criterion individually first and then using all the features found independently of the criterion used.

#### Visual Classification

**Fig. 4:**
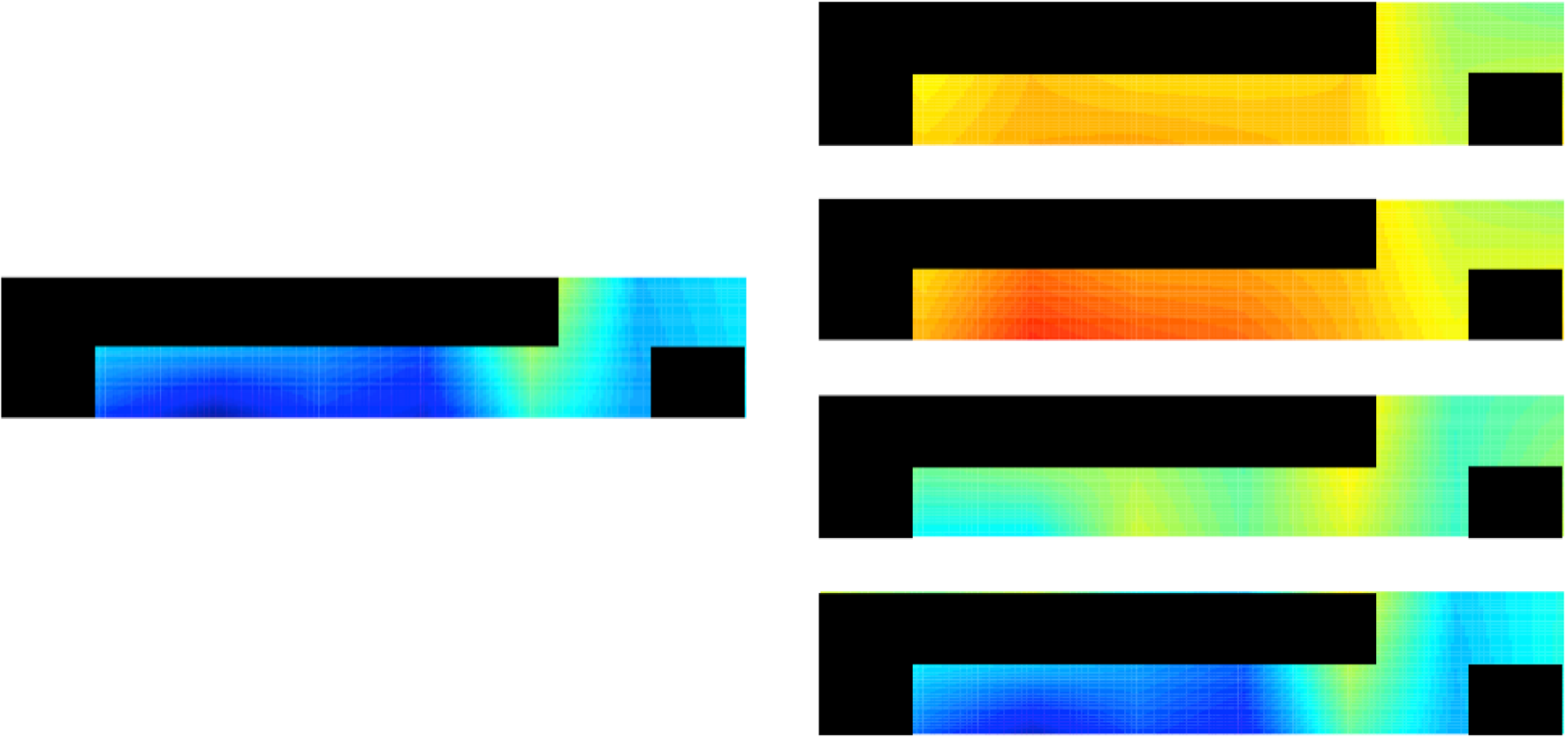
User interface used to classify every question as one of four classes. Non-relevant voxels are colored in black

$\overline{\Delta B}_{v}^{q}$ (Eq. (3)) was mapped into the form of a topogram, represented as a false color map using a linear interpolation at the edges of each color. Only the relevant voxels selected in the previous section are shown to the evaluator. The rest are blocked out by coloring them in black, as shown in Fig. 4. The minimum value of $\overline{\Delta B}_{v}^{q}$ is represented in blue, while the maximum value is represented in red. The same process was used for $\overline{\Delta B}_{v}^{m}$, $\overline{\Delta O}_{v}^{q}$, and $\overline{\Delta O}_{v}^{m}$ (Eq. (4)).

**Fig. 5:**
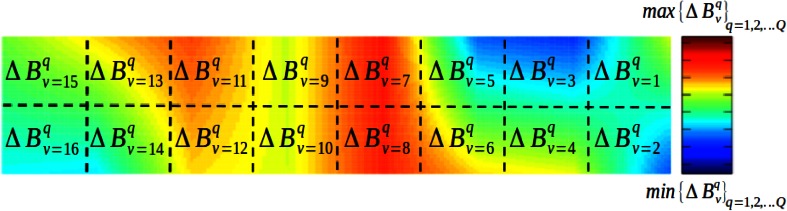
Topogram of $\overline{\Delta B}_{v}^{q}$, created by mapping Eq. (3) into false color space. Analogous topogram is created for every question in questionnaire

This results in *Q* + *M* topograms for each subject. A single topogram is shown in Fig. 5. The topogram mappings of $\overline{\Delta B}_{v}^{m}$ are computed and taken as visual references. The topogram of each question ($\overline{\Delta B}_{v}^{q}$) is compared against the visual references and labeled as the category that it resembles the most. It should be noted that the reference topograms are mapped for each subject individually.

This process was performed for the 30 questions for each of the 10 subjects, which were presented in random order to the evaluator, who had normal vision acuity, was not color-blind, and had no history of ophthalmic diseases that would prevent him from evaluating the results correctly.

#### Automatic Classifiers

An SVM classifier was used to perform automatic classification. This learning machine non-linearly maps input vectors into a very-high-dimensional feature space. In this new space, the different classes can be separated [16]. The shape of the decision border depends on the parameters used (especially the kernel type). These borders can be linear or highly non-linear. The basic idea of the SVM is the construction of an optimal hyperplane that separates different classes with the maximal margin, which is defined as the distance between the hyperplane and the closest training input vector [17]. The LIBSVM library was used to train the classifier as suggested in a previous study [18].

The performance of the visual and automatic classifiers was evaluated in terms of specificity, sensitivity, and accuracy. Specificity is defined as the ratio of true negatives to the sum of false positives and true negatives; sensitivity is the ratio of true positives to the sum of true positives and false negatives; finally, accuracy is the ratio of the sum of true positives and true negatives to the sum of true positives, true negatives, false positives, and false negatives.

## Results and Discussion

### Feature Selection for Visual Classification

Table 1 shows the voxels selected using the various criteria. Only the relative change in blood volume presented relevant differences among classes. Additionally, an analysis of the results of the three criteria indicates that, with our dataset, it is not possible to discriminate between Non-Induced Truths and Non-Induced Lies. Therefore, the performance of classifiers in a three-class problem (Induced Lies, Induced Truths, Non-Induced responses) was compared using exclusively the relative changes in blood volume [19].

**Table 1:** Relevant voxels used to discriminate among classes using relative change in blood volume

Method	Description	Relevant Voxels
Heuristic criterion	Discrimination between non-induced truths and non-induced lies	V8, V10
Heuristic criterion	Discrimination between induced truths and induced lies	V6, V8, V12
Non-parametric criterion	Discrimination between non-induced truths and non-induced lies	V10, V12
Non-parametric criterion	Discrimination between induced truths and induced lies	V1, V8
Parametric criterion	Discrimination between non-induced truths and non-induced lies	–
Parametric criterion	Discrimination between induced truths and induced lies	V1, V8
Parametric criterion	Discrimination between induced and non-induced responses	V3, V14

### Classification

Table 2 shows the performance of the human evaluator when classifying the responses as Induced Lies, Induced Truths, and Non-Induced responses. The non-parametric and heuristic criteria showed similar performance in terms of accuracy (85.33 and 84.00 %, respectively). The parametric criterion achieved only 81.67 %. The decrease in performance was expected because this criterion assumes a normal distribution in the data, while the non-parametric criterion does not make that assumption. Although the non-parametric and heuristic criteria used only 4 voxels, the addition of more voxels using the combination of the three feature selection methods did not improve performance, since the use of the three criteria combined achieved an accuracy of 84.00 %.

**Table 2:** Accuracy of evaluator in 3-class problem using visual classification

Subject	Heuristic criterion (%)	Non-parametric criterion (%)	Parametric criterion (%)	3 criteria (%)
S. 1	86.67	83.33	83.33	86.67
S. 2	76.67	73.33	73.33	76.67
S. 3	93.33	93.33	100	100
S. 4	80.00	90.00	73.33	70.00
S. 5	80.00	76.67	86.67	80.00
S. 6	96.67	90.00	90.00	86.67
S. 7	53.33	60.00	56.67	63.33
S. 8	93.33	96.67	93.33	96.67
S. 9	83.33	86.67	70.00	80.00
S. 10	96.67	93.33	90.00	100
Av.	84.00	84.33	81.67	84.00

Performance was computed using voxels selected by each feature selection criterion individually and using voxels selected by 3 criteria together

When the problem was simplified to discriminate between induced and non-induced responses, the performance of classification improved, as can be seen in Table 3. In this case, all the feature selection methods showed similar performance. The combination of the three criteria showed the best results, with an accuracy of 92.00 %, followed by the parametric and non-parametric criteria (91.00 % each one) and finally the heuristic criterion (90.33 %).

**Table 3:** Accuracy of evaluator in bi-class problem using visual classification

Subject	Heuristic criterion (%)	Non-parametric criterion (%)	Parametric criterion (%)	3 criteria (%)
S. 1	96.67	96.67	96.67	96.67
S. 2	90.00	90.00	90.00	90.00
S. 3	100	100	100	100
S. 4	86.67	93.33	93.33	86.67
S. 5	90.00	90.00	93.33	93.33
S. 6	100	96.67	100	100
S. 7	60.00	66.67	70.00	73.33
S. 8	93.33	96.67	100	96.67
S. 9	90.00	86.67	73.33	83.33
S. 10	96.67	93.33	93.33	100
Av.	90.33	91.00	91.00	92.00

Performance was computed using voxels selected by each feature selection criterion individually and using voxels selected by 3 criteria together

Table 4 shows the performance of the SVM classifier for the same classification task. For this case, a fifth feature set consisting of all 16 voxels was added. The SVM classifier took advantage of the increase in the amount of data, since the best results were obtained using the 16 available voxels (95.63 %), followed by the use of the three criteria together (which selected 7 voxels and achieved an accuracy of 93.22 %) and finally the heuristic, non-parametric, and parametric criteria (each of which selected 4 voxels and had accuracies of 91.62, 89.41, and 87.82 % respectively).

**Table 4:** Accuracy of SVM classifier using different feature sets in three-class problem

Subject	Heuristic criterion (%)	Non-parametric criterion (%)	Parametric criterion (%)	3 criteria (%)	All voxels (%)
S. 1	91.44	79.22	90.66	92.66	91.66
S. 2	90.33	67.77	69.33	88.66	93.44
S. 3	97.55	97.77	97.88	98.00	98.44
S. 4	79.77	89.88	91.44	89.55	91.88
S. 5	97.00	94.22	95.11	97.55	99.77
S. 6	93.11	89.55	90.22	96.33	99.22
S. 7	84.66	87.11	64.33	80.77	87.22
S. 8	92.11	96.11	93.11	96.11	98.55
S. 9	92.11	95.88	90.33	94.88	98.77
S. 10	98.11	96.55	95.77	97.66	97.33
Av.	91.62	89.41	87.82	93.22	95.63

Finally, the ability of every set of features to predict the elements of an individual class was compared. Each method was compared in terms of specificity, sensitivity, and accuracy. Table 5 shows the performance of each set. The table compares the performance achieved when using the set of features selected by the heuristic, non-parametric, and parametric criteria, the three criteria together, and the information of all the voxels for both SVM and visual classification (Vis). For the visual approach, the heuristic criterion should be used for identifying Induced Lies and Induced Truths (it presented the highest sensitivity scores of 82.4 and 87.0 %, respectively), while the three criteria (sensitivity score of 87.2 %) should be used for identifying Non-Induced responses. Otherwise, the use of all the available voxels is suggested.

**Table 5:** Specificity (Spc.), Sensitivity (Sen.), and Accuracy (Acc.) averaged across all subjects when identifying elements of a single class

	Spc.		Sen.		Acc.	
	Vis. (%)	SVM	Vis.	SVM	Vis.	SVM
Non-induced responses						
Heuristic	94.7	94.8	82.5	89.6	90.3	93.1
Non-parametric	94.8	93.6	84.1	93.2	91.0	93.5
Parametric	93.9	93.5	85.4	91.9	91.0	93.0
3 criteria	94.4	95.7	87.2	93.9	92.0	95.1
All voxels	–	97.7	–	95.9	–	97.1
Induced lies						
Heuristic	88.0	93.2	82.4	88.7	86.3	91.7
Non-parametric	87.4	93.8	79.5	85.3	85.0	90.8
Parametric	88.1	92.8	78.3	84.1	85.0	89.8
3 criteria	88.7	95.3	80.2	91.5	86.0	94.0
All voxels	–	96.6	–	93.8	–	95.6
Induced truths						
Heuristic	93.5	99.0	87.0	95.3	91.3	97.7
Non-parametric	93.0	96.5	86.0	89.8	90.6	94.2
Parametric	90.5	95.3	81.0	87.8	87.3	92.7
3 criteria	92.9	98.9	84.3	94.6	90.0	97.4
All voxels	–	98.7	–	96.5	–	98.0

The SVM classifier outperformed the visual classification independently of the feature set, and the best results were obtained when using the relative changes in blood volume in all the available voxels; however, this also involves a higher computational cost. These results suggest that it is possible to use a visual method to classify the subject responses without the need for an automatic classifier, but with a performance decrease of about 11.3 % (the difference between the best results achieved by the SVM classifier compared with those of the human evaluator in the tri-class problem). Whether the decrease in computational cost is worth the decrease in performance is a decision that must be made by the interviewer. These results suggest that it is possible not only to identify a general pattern in the hemodynamic behavior when the subjects are lying or telling the truth, as presented in a previous study [9], but also to classify each response individually.

**Fig. 6:**
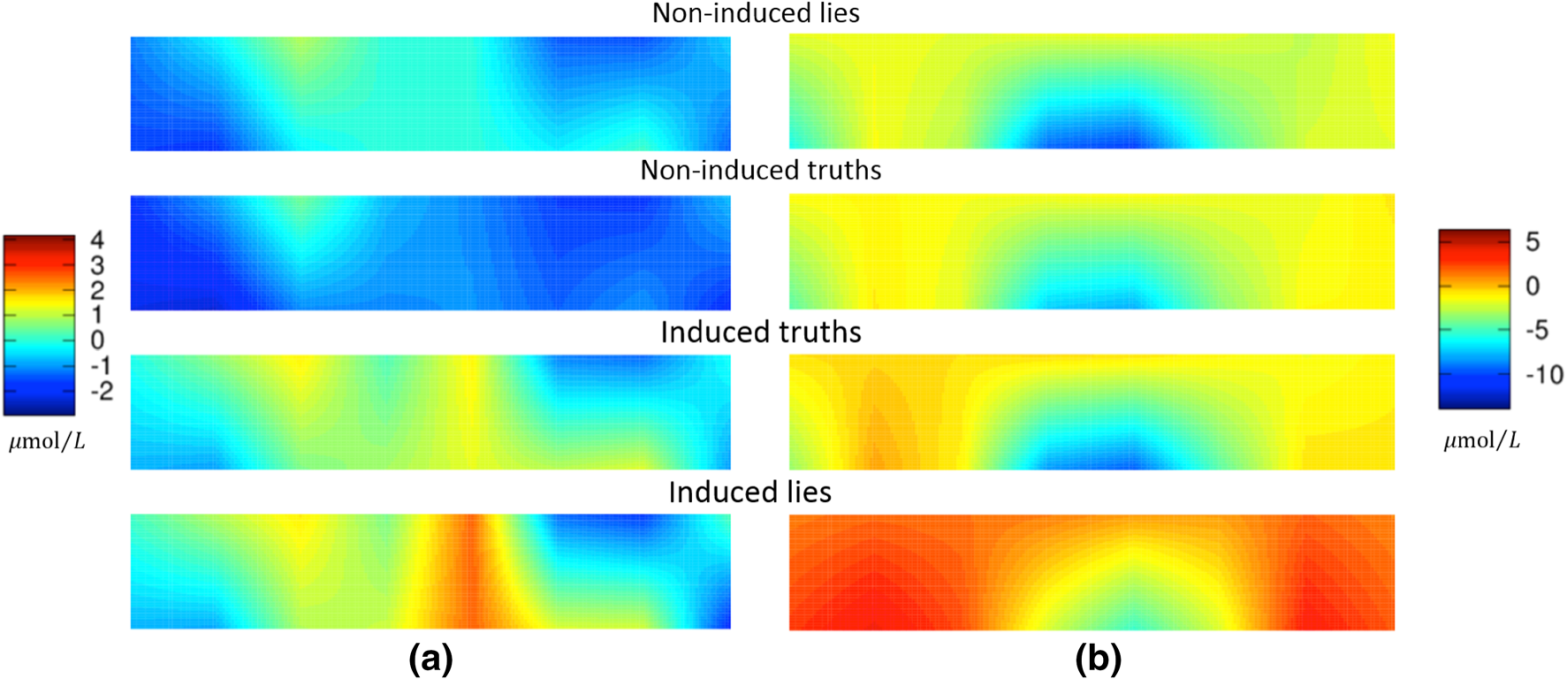
Characteristic images of four classes for **a** Subject 1 and **b** Subject 7. It is difficult to visually discriminate among classes 1, 2, and 3 for Subject 7; however, the difference is clearer for Subject 1

Regardless of the feature selection criteria and the classification method, important variations are present in the performance scores among different subjects. This is explained by the difference among classes being very clear for some subjects, but very subtle for others. Figure 6 shows a comparison of the characteristic image of each class for two subjects.

**Fig. 7:**
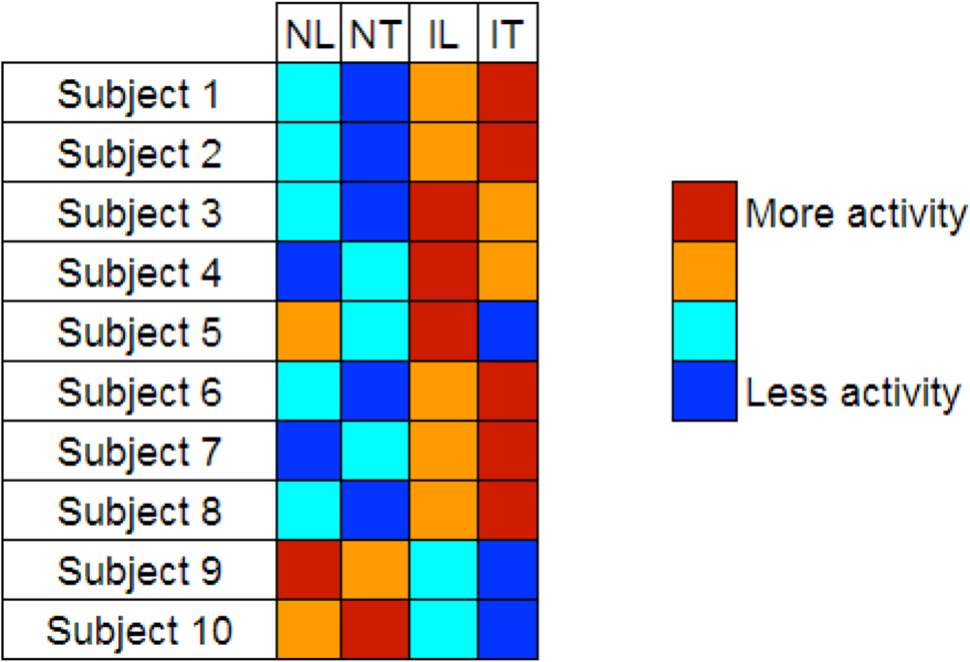
Relative level of brain activity required to tell Non-Induced Lies (NL), Non-Induced Truths (NT), Induced Lies (IL), and Induced Truths (IT) using information of relative changes in blood volume

The relative activity level for each category (Induced Lies, Induced Truths, Non-Induced Lies, and Non-Induced Truths) was computed as the average of all the voxels *v*_*r*_ for the questions within a given category. Figure 7 shows these levels for all the subjects. In general, there is a difference among subjects when telling Induced and Non-Induced responses (the two highest values in the false color scale are either the induced responses or the non-induced responses in all the subjects but Subject 5). Figure 7 also shows that the brain activity in most subjects is greater when telling a Non-Induced Lie than when telling a Non-Induced Truth. This result is consistent with a previously study [10], where the authors found that telling a lie induces greater brain activation, as indicated by an increment in the blood volume in the prefrontal cortex. The behavior is different for the induced responses. For these, half of the subjects presented greater activity when telling an Induced Lie, and the other half presented greater activity when telling an Induced Truth.

When a human performs the classification, it is important that he/she is able to visually distinguish between classes. For this reason, a general visual pattern that represents the average response of all the subjects is ideally computed and showed as a topogram. This serves as reference to classify each response. However, we were not able to find a general pattern that represented an average response, but we were able to find individual visual patterns representing the average response for each subject. This allowed classifying each response with the previously mentioned accuracy.

## Conclusion

A comparison between a human evaluator and an automatic classifier for deception detection based on brain hemodynamics from fNIRS was presented. The human evaluator used a visual approach for classification. For this, topograms of the activity in the relevant voxels of the prefrontal cortex were constructed. Relevant voxels were selected based on parametric, non-parametric, and heuristic criteria. With the proposed methodology, the human evaluator successfully identified 84.33 % of the answers in a tri-class problem (Induced Lies, Induced Truths, and Non-Induced responses) and 92 % of the answers in a bi-class problem (Induced Lies and Induced Truths). In comparison, an SVM classifier correctly classified 95.63 % of the answers in the tri-class problem using the same relevant features; however, the improvement in performance comes at a cost of higher computational complexity. The selection of the approach should be determined by the application: if the goal is to classify the subject’s responses while the interviewer is executing the questionnaire with fair performance, the visual approach is suggested. On the other hand, if the goal is to classify the subject’s responses with high accuracy, the automatic classifier is suggested.
